# Percutaneous nephrolithotomy versus flexible ureteroscopic lithotripsy in the treatment of upper urinary tract stones: a meta-analysis comparing clinical efficacy and safety

**DOI:** 10.1186/s12894-020-00677-4

**Published:** 2020-07-25

**Authors:** Yeda Chen, Yaoan Wen, Qingfeng Yu, Xiaolu Duan, Wenqi Wu, Guohua Zeng

**Affiliations:** Department of Urology, Minimally Invasive Surgery Center, The First Affiliated Hospital of Guangzhou Medical University, Guangdong Key Laboratory of Urology, Kangda Road 1#, Haizhu District, Guangzhou, 510230 Guangdong China

**Keywords:** Percutaneous nephrolithotomy, Ureteroscopic lithotripsy, Upper urinary tract stones, Efficacy, Safety

## Abstract

**Background:**

Upper urinary tract stones is the most common diseases in urology. Percutaneous nephrolithotomy (PCNL) and ureteroscopic lithotripsy (fURL) are common treatment, but both their efficacy and safety are controversial. Thus we aim to evaluate the efficacy and safety of PCNL and fURL in the treatment of upper urinary tract stones, providing a reference for clinical work.

**Methods:**

PubMed, Web of Science, Embase and CNKI were searched through Apr. 1, 2019 to identify eligible studies. Data were analyzed by using RevMan 5.3 and Stata 12.0 software. Pooled relative risks (RRs) or weighted mean difference (WMD) with 95% confidence intervals (CIs) were calculated using fixed or random effects methods. Publication bias and sensitivity analysis were performed.

**Results:**

Four randomized controlled trials (RCTs), fifteen cohort studies involving 1822 patients were included. Stone-free rate of PCNL was significantly high than that of fURL (RR: 1.07; 95% CI: 1.03, 1.12; *P* = 0.0004). The decline of hemoglobin in PCNL was significantly high than that of fURL (WMD: 1.07; 95% CI: 0.54, 1.61; *P* < 0.0001). The number of blood transfusion was significantly greater in the PCNL compared to the fURL (RR: 5.04; 95% CI: 1.78, 14.24; *P* = 0.002). The incidence of postoperative bleeding or hematuria showed greater significantly difference in the PCNL compared to the fURL (RR: 2.72; 95% CI: 1.55, 4.75; *P* = 0.0005). Operation time, fever, infection, perforation, requiring drug analgesia was not significantly different between two surgical procedures.

**Conclusions:**

In the treatment of upper urinary tract stones, the stones clearance rate of PCNL is higher than fURL, and the safety of fURL is higher than PCNL.

## Background

Upper urinary tract stones, including kidney stones and ureteral stones, are the most common diseases in urology clinical workers. For the treatment of upper urinary calculi, in the past, opening incision and stone removal were often used. The surgical injury was large, the complication rate was high, and the patient recovered for a long time. In recent years, with the development of medical equipment and medical technology, extracorporeal shock wave lithotripsy (ESWL), retrograde ureteroscopic lithotripsy (RIRS), percutaneous nephrolithotomy (PCNL) and other minimally invasive or non-invasive treatment methods appear successively [1, 2]. Minimally invasive surgery is usually chosen after ESWL treatment is ineffective. PCNL, microchannel PCNL (mPCNL), ultra-fine passage percutaneous kidney mirror lithotripsy (UMP) and ultra-microchannel percutaneous nephrolithotomy (SMP) have become one of the preferred methods for clinical treatment of upper urinary calculi with its minimally invasive and high-efficiency advantages. The appearance of ureteroscopy (fURL) had a huge impact on the treatment of upper urinary tract stones. Compared with ureteral hard and percutaneous nephrolithoscopy, it has a flexible lens body. Even if the stones are in the kidney, moving or descending into the ureter can also effectively crush the stone [3], thus having considerable advantages.

In the guidelines issued by the American Urological Association (AUA), fURL treatment is recommended for kidney stones < 2 cm in diameter, and PCNL is recommended for upper urinary calculi > 2 cm in diameter and more complicated stones. Jacquemet [4] et al. compared the calculus clearance rate and complication rate of 371 cases of renal calculi in different sites by fURL. It was found that there was no difference in the efficacy of fURL in the treatment of renal calculi and other renal stones. Professor Cheng [5] used fURL to treat staghorn calculi and achieved good results, and proposed that fURL can handle all the stones that PCNL can handle, and it is expected to replace high-risk surgery such as PCNL in the future. It has been reported in the literature that PCNL and fURL are both feasible and effective methods in the current comparative study of medium and large renal stone treatments [6].

Both procedures are the first-line method for the treatment of upper urinary calculi. Therefore, it is quite meaningful to compare the efficacy and safety of the two surgical procedures. At present, the clinical reports on the efficacy and safety of upper urinary calculi are mostly single-center, small-sample clinical controlled studies, and evidence-based medical evidence is lacking. We collect a clinical controlled study of these two surgical procedures for upper urinary calculi, using meta-evidence medical analysis, comprehensive analysis and re-evaluation of published clinical controlled trials, in order to more scientifically evaluate their effectiveness and safety, providing a reference for clinical work.

## Methods

A literature-search strategy, inclusion and exclusion criteria, outcome measurements, and methods of statistical analysis were prepared a priori according to the Preferred Reporting Items for Systematic Reviews and Meta-analysis (PRISMA) guidelines and the Meta-analysis of Observational Studies in Epidemiology Guidelines. Our study was based on data from previously published studies. Therefore, patients’ consent or ethical approval were not necessary.

### Literature search

A comprehensive literature retrieval was guided by independently by two investigators in Web of Science databases, PubMed, Embase and China National Knowledge Infrastructure databases (CNKI) with a cutoff date of Apr. 1, 2019. The following MeSH terms were used in search strategy: (“flexible ureteroscopic lithotripsy” OR “FURL” OR “RIRS” OR “percutaneous nephrolithotomy” OR “PCNL” OR “PNL”) AND (“ureteral calculus” OR “Upper ureteralstones” OR “renal stones”). Besides, the references of other related articles were also hand-searched for additional eligible studies.

### Inclusion and exclusion criteria

Studies meeting the predetermined criteria were included: (1) patients with large (> 10 mm) proximal ureteral stones and accompanied with secondary renal stones (< 10 mm); (2) comparing PCNL(or mPCNL) and fURL; (3) both surgical techniques should be performed on adults; (4) the full text could be accessed online; (5) reporting at least one of clinical outcomes of interest (described in data extraction part). Exclusion criteria included: (1) only one procedure, no comparison study; (2) subjects < 18 years old; (3) repeated publications, select the latest published time; (4) a literature is less information so that data can not be obtained.

### Data extraction and quality assessment

We screened the studies on the basis of inclusion and exclusion criteria. Two investigators independently extracted and reviewed the data from the eligible studies. Any disagreement was verified with a third investigator to resolve the dispute. Through a same standardized information collection table, the following data were extracted from all eligible studies: first author’s name, publication year, country, primary site, study design, surgical technique, age, sex, stone burden, number of cases, stone free rate, operative time, decline of hemoglobin (Hb), blood transfusion, bleeding/hematuria, fever/infection, perforation, requirements of drug analgesia for in the statistical analysis.

The quality of eligible studies was performed by two investigators using the Newcastle-Ottawa Scale (NOS) [7] for cohort studies. This tool comprises three broad categories (selection of controls and cases, comparability and outcomes of study participants), and scores of 0–4, 0–2, and 0–3 are assigned for these three categories, respectively. Studies with final scores of 0–3, 4–6, and 7–9 were represented as low, moderate, and high quality, respectively. This quality score was used to measure the strength of each study’s evidence. The Cochrane Collaboration’s tool was used to assess the risk of bias of randomized controlled trials (RCTs).

### Data synthesis and statistical analysis

All analyses were performed using RevMan 5.3 and Stata 12.0 software of the Cochrane Collaboration to compare the safety and efficacy of PCNL and fURL. Relative risk or odds ratio was used for dichotomous data, and weighted or standardized mean difference was used for the continuous data. All the outcomes were reported with 95% confidence intervals (95% CI). Cochran’s test and *I*^*2*^ statistic were used to assess the heterogeneity among studies [8]. When *I*^*2*^ < 50% or *P* > 0.05 were considered as no heterogeneity and a fixed-effects model was performed. For significant heterogeneity among studies (*I*^*2*^ ≥ 50% or *p* ≤ 0.05), the random-effects model was applied. The funnel plot with Begg’s correlation test and Egger’s linear regression test were applied to assess the potential publication bias. The stability of the results was assessed with the sensitivity analysis.

## Results

### Literature retrieval and analysis

**Fig. 1:**
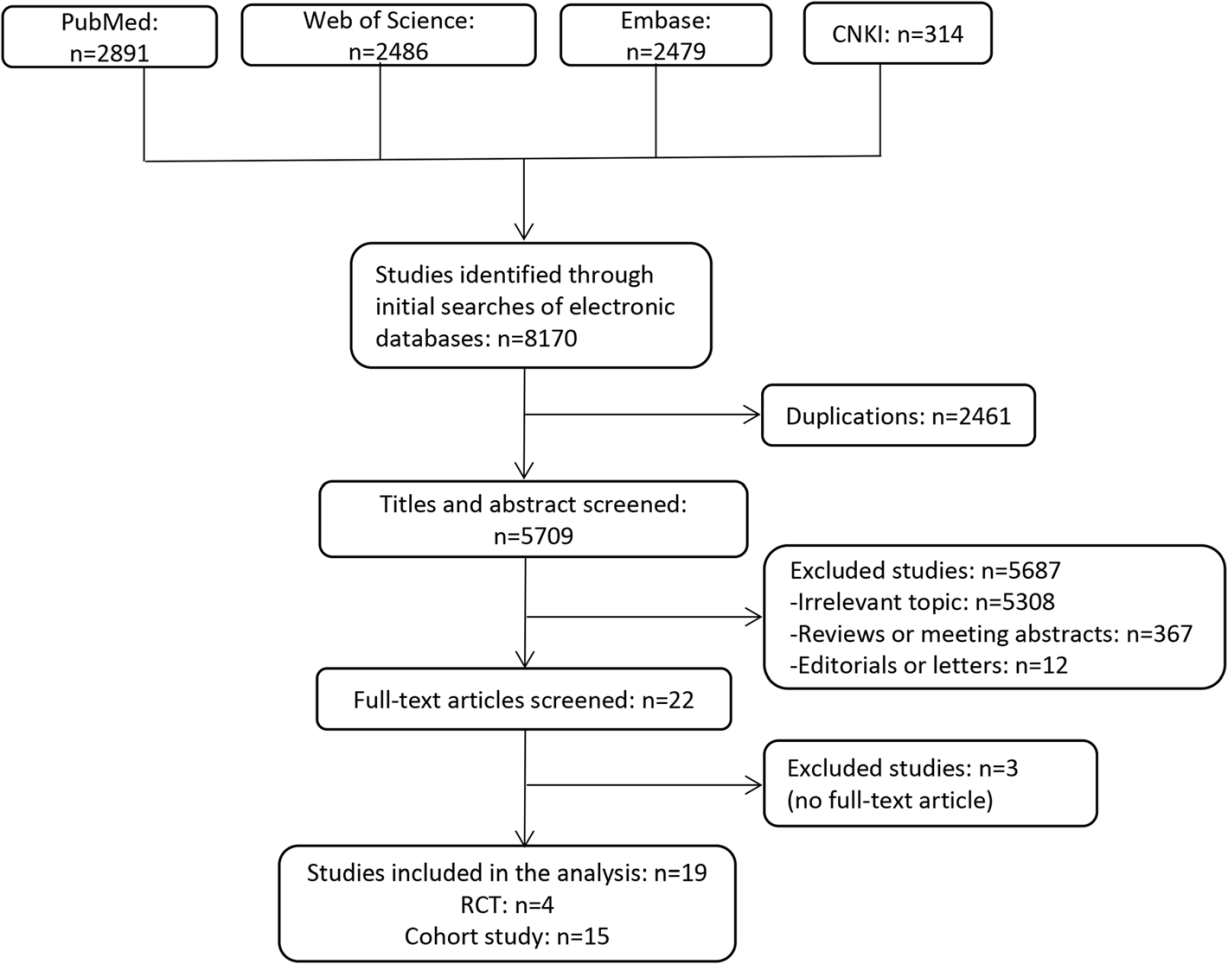
Flow diagram of the literature search and selection

A flowchart of the literature search and study selection was shown in Fig. 1. A total of 8170 potential studies were found from the Embase, PubMed, Web of Science databases and CNKI. Based on the above-mentioned inclusion and exclusion criteria, a total of 19 studies were identified in the meta-analysis at last. A summary of the main characteristics of the included studies published from 2008 to 2017 was shown in Table 1, including 4 RCTs [8], 15 cohort studies [9–13, 15–17, 19–22, 24–26]. The 19 studies included 1822 individuals. Basic characteristics, such as age, sex ratio, stone burden and stone side, were described comparable between PCNL and fURL group in each study and the data was presented.

**Table 1:** Main characteristic of the included studies

Reference, year	Nation	primary site	Study design	Surgical technique	Sample size	Age (year)		Sex (M/F)	Stone burden		Stone side
						Mean ± SD	Median ± range		(Mean ± SD)	Median ± range	(left / right)
Aboutaleb, 2012 [9]	Kuwait	Lower calyx	CS	PCNL	19	45.33 ± 14.30	–	14/5	17.30 ± 3.30	–	ND
				fURL	13	47.20 ± 15.20	–	7/6	14.50 ± 3.20	–	ND
Armagan, 2015 [10]	Turkey	Kidney	CS	mPCNL	68	43.60 ± 18.90	–	35/33	13.70 ± 4.20	–	ND
				fURL	59	49.30 ± 15.30	–	36/23	14.40 ± 3.10	–	ND
Bozkur, 2011 [11]	Turkey	Lower calyx	CS	PCNL	42	47.40 ± 15.50	–	25/17	1.70 ± 0.12	–	20/22
				fURL	37	41.20 ± 13.60	–	21/16	1.65 ± 0.69	–	19/18
Chung, 2008 [12]	America	Kidney	CS	PCNL	15	–	58.00^a^	40/60	–	1.80 (1.0–2.0)	60/40
				fURL	12	–	58.50^a^	58/42	–	1.25 (1.0–1.9)	58/42
Ferroud, 2011 [13]	French	Kidney	CS	mPCNL	101	51.70 ± 16.10	–	80/21	8.90 ± 2.70	–	ND
				fURL	43	49.20 ± 14.80	–	28/15	8.50 ± 3.20	–	ND
Gu, 2013 [14]	China	Upper ureter	RCT	mPCNL	30	42.50 ± 10.10	–	17/13	17.27^a^	–	16/14
				fURL	29	44.22 ± 13.00	–	18/11	16.23^a^	–	12/17
Hu, 2016 [15]	China	ureter	CS	mPCNL	104	65.50 ± 4.90	–	56/48	15.80 ± 3.40	–	53/51
				fURL	80	65.10 ± 5.20	–	45/35	15.80 ± 3.40	–	47/33
Kirac, 2013 [16]	Turkey	Lower calyx	CS	mPCNL	37	41.02 ± 10.30	–	25/12	10.50 ± 2.20	–	16/22
				fURL	36	37.80 ± 8.70	–	22/14	10.20 ± 2.90	–	14/22
Kruck, 2013 [17]	Germany	Kidney	CS	mPCNL	172	53.30 ± 14.80	–	109/63	12.60 ± 9.50	–	ND
				fURL	108	50.00 ± 16.70	–	69/39	6.80 ± 6.90	–	ND
Kumar, 2015 [18]	India	Lower calyx	RCT	mPCNL	41	33.70 ± 1.60	–	20/21	13.30^a^	–	22/19
				fURL	43	33.40 ± 1.40	–	20/23	13.10^a^	–	22/21
Lee, 2015 [6]	Korea	Kidney	RCT	mPCNL	35	59.30 ± 13.30	–	28/7	39.10^a^	–	21/14
				fURL	33	55.80 ± 11.20	–	28/5	28.90^a^	–	23/10
Ozgor, 2016 [19]	Turkey	Kidney	CS	mPCNL	56	51.40 ± 14.30	–	25/31	19.50 ± 3.90	–	25/31
				fURL	56	54.20 ± 10.60	–	22/34	18.30 ± 3.20	–	37/19
Ozgor, 2018 [20]	Turkey	Kidney	CS	mPCNL	58	66.90 ± 5.90	–	28/30	20.30 ± 5.60	–	ND
				fURL	60	67.70 ± 6.70	–	33/27	19.00 ± 4.50	–	ND
Pan, 2013 [21]	China	Kidney	CS	mPCNL	59	49.37 ± 14.20	–	36/20	22.37 ± 2.70	–	23/36
				fURL	56	49.32 ± 13.70	–	37/22	22.28 ± 2.60	–	30/26
Sabnis, 2012 [22]	India	Kidney	CS	mPCNL	32	44.48 ± 12.36	–	19/13	1.52 ± 0.33	–	10/22
				fURL	32	49.28 ± 12.19	–	25/7	1.42 ± 0.34	–	16/16
Sabnis, 2013 [23]	India	Kidney	RCT	mPCNL	35	38.60 ± 14.60	–	22/13	11.00^a^	–	16/19
				fURL	35	43.70 ± 12.10	–	24/11	10.40^a^	–	18/17
Schoenthaler,2015 [24]	Germany	Kidney	CS	UMP	30	–	54.30 (19–72)	17/13	–	15.10 (10–20)	ND
				fURL	30	–	56.30 (18–76)	ND	–	14.40 (10–20)	ND
Wilhelm, 2015 [25]	Germany	Kidney	CS	UMP	25	–	51.56 (15–75)	15/10	–	19.28 (10–35)	ND
				fURL	25	–	51.36 (19–77)	19/6	–	19.20 (10–35)	ND
Zhang, 2014 [26]	China	Upper ureter	CS	mPCNL	32	42.70 ± 13.60	–	24/8	15.60 ± 2.50	–	ND
				fURL	44	43.30 ± 11.00	–	29/15	14.90 ± 2.30	–	ND

*RCT* randomized controlled trial, *CS* cohort study, *PCNL* percutaneous nephrolithotomy, *mPCNL* minipercutaneous nephrolithotomy, *fURL* flexible ureteroscopic lithotripsy, *ND* not demonstrated

^a^No SD or range was demonstrated in primary studies

### Quality of the studies

**Fig. 2:**
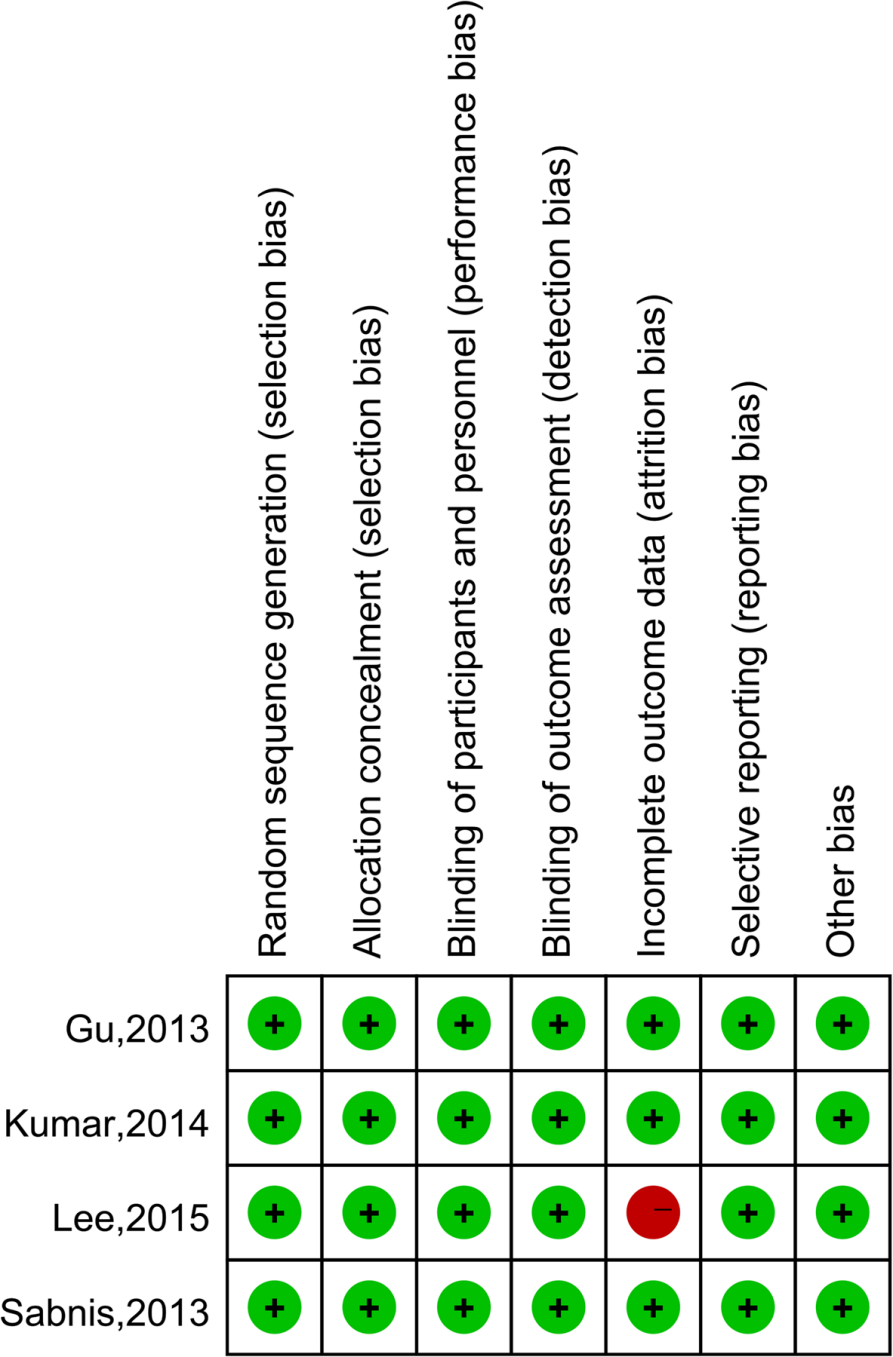
Risk of bias for randomized controlled trials

Figure 2 showed that all of the RCTs described suggested randomization. One study [9–13, 15–17, 19–22, 24–26] failed to collect complete outcome data. It was quite difficult to get a accurate result, so a high risk of bias was judged in this part for this study. Other studies had low risk of selection bias, performance bias, detection bias, attrition bias, reporting bias and other bias. Therefore, three RCTs [14, 18, 23] were judged to be of high quality. The quality of 15 cohort studies was assessed using the NOS. As shown in Table 2, the highest quality score was 9 and the lowest was 6. The average quality score for all cohort studies was 7.3. As a result, the cohort studies were considered high quality.

**Table 2:** Quality assessment of included cohort studies

Source	Selection				Comparability	Outcome			Scores
Author, year	Representativeness of the exposed cohort	Selection of the non exposed cohort	Ascertainment of exposure	Demonstration that outcome was not present at start of study	select the most important factor^a^	Assessment of outcome	Follow-up long enough	Adequacy of follow up of cohorts	
Aboutaleb,2012 [9]	–	–	★	★	★★	★	★	★	7
Armagan,2015 [10]	–	–	★	★	★★	★	–	★	6
Bozkur,2011 [11]	★	★	★	★	★★	★	★	★	9
Chung,2008 [12]	–	–	★	★	★★	★	★	★	7
Ferroud,2011 [13]	–	–	★	★	★★	★	★	★	7
Hu, 2016 [15]	★	★	★	★	★★	★	★	★	9
Kirac,2013 [16]	★	★	★	★	★★	★	★	★	9
Kruck,2013 [17]	–	–	★	★	★★	★	★	★	7
Ozgor,2016 [19]	–	–	★	★	★★	★	★	★	7
Ozgor, 2018 [20]	–	–	★	★	★★	★	★	★	7
Pan, 2013 [21]	–	–	★	★	★★	★	★	★	7
Sabnis,2012 [22]	–	–	★	★	★★	★	★	★	7
Schoenthaler,2015 [24]	★	★	★	★	★★	★	–	★	8
Wilhelm,2015 [25]	–	–	★	★	★★	★	–	★	6
Zhang,2014 [26]	–	–	★	★	★★	★	–	★	6

^a^A maximum of two stars can be awarded for select the most important factor or additional factor

### Stone free rate

**Fig. 3:**
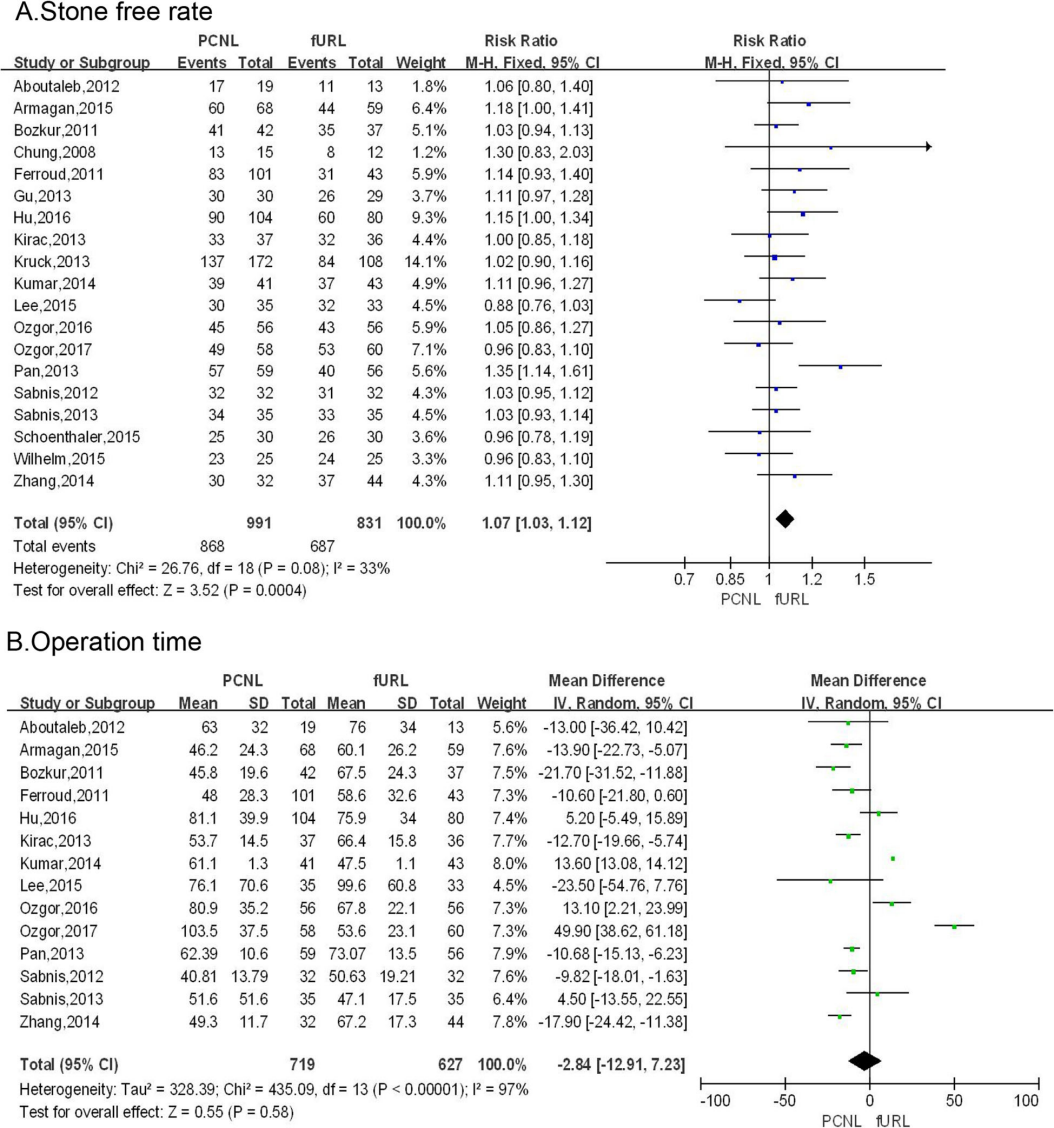
Forest plots of (**a**) stone free rate, (**b**) operative time

Pooling the data from 19 studies [6, 9–26] demonstrated that the stone-free rate of PCNL group was significantly high than that of fURL group (RR: 1.07; 95% CI: 1.03, 1.12; *P* = 0.0004; Fig. 3a).

### Operation time

Pooling the data from 14 studies [6, 9–26] that assessed operation time showed no significant difference between PCNL group and fURL group (WMD: − 2.84 min; 95% CI: − 12.91, 7.23; *P* = 0.58; Fig. 3b).

### Operative complications

#### Decline of HB

**Fig. 4:**
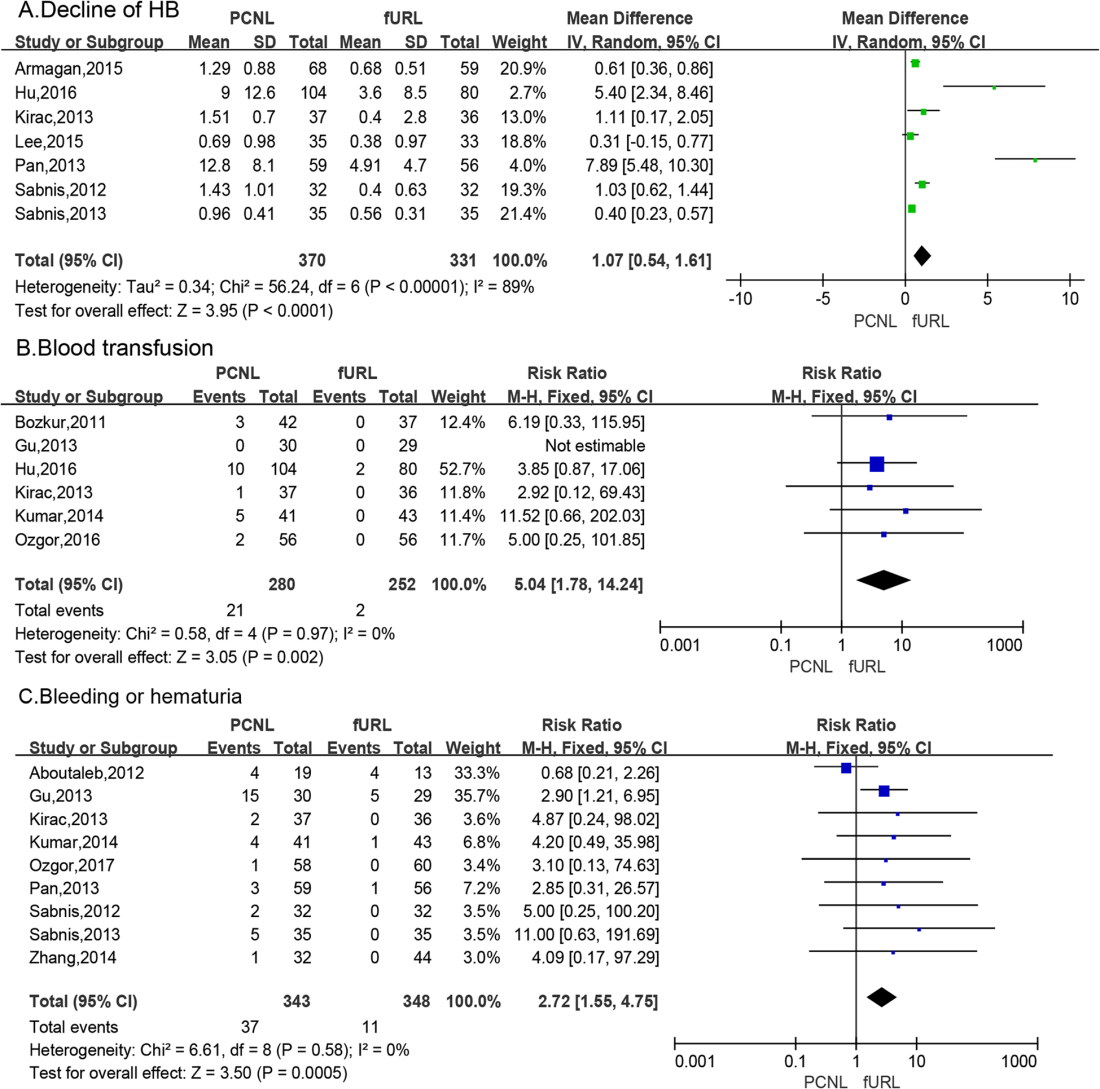
Forest plots of (**a**) decline of Hb, (**b**) blood transfusion,(**c**) bleeding or hematuria

Meta-analysis of 7 studies [6, 10, 15, 16, 21–23] by a random effects model showed that the decline in PCNL group was significantly high than that of fURL group (WMD: 1.07; 95% CI: 0.54, 1.61; *P* < 0.0001; Fig. 4a).

#### Blood transfusion

Meta-analysis of 6 studies [6, 10, 15, 16, 21–23] by a fixed effects model showed that the number of blood transfusion was significantly greater in the PCNL group compared to the fURL group (RR: 5.04; 95% CI: 1.78, 14.24; *P* = 0.002; Fig. 4b).

#### Bleeding or hematuria

Pooling the data from 9 studies [9, 14, 16, 18, 20–23, 26] that assessed the incidence of postoperative bleeding or hematuria showed significantly greater difference in the PCNL group compared to the fURL group (RR: 2.72; 95% CI: 1.55, 4.75; *P* = 0.0005; Fig. 4c).

#### Fever or infection

**Fig. 5:**
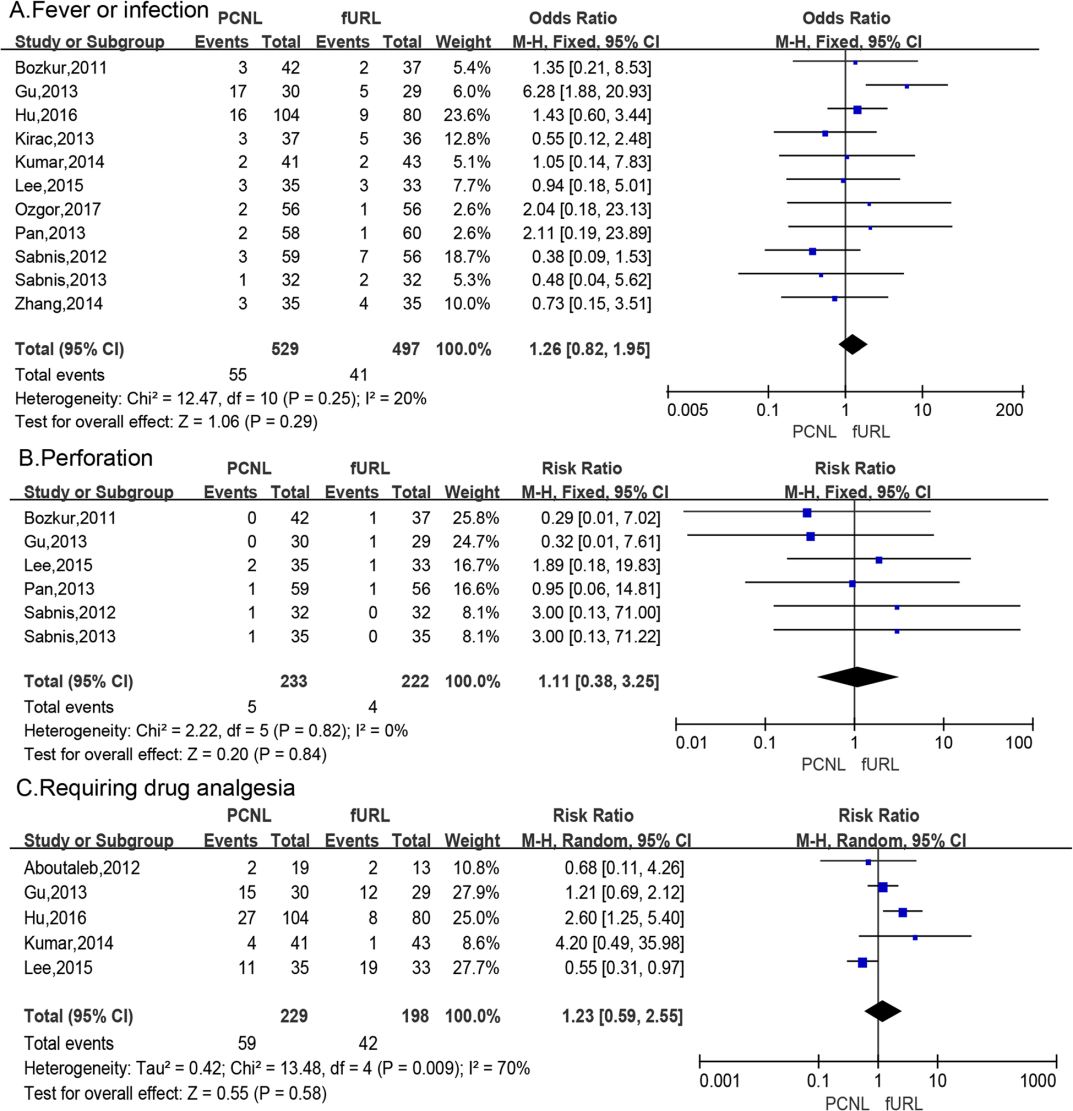
Forest plots of (**a**) fever or infection, (**b**)perforation,(**c**) requiring drug analgesia

Meta-analysis of 11 studies [6, 11, 14–16, 18, 20–23, 26] by a fixed effects model showed the incidence of postoperative fever or infection was not signifcantly different in the PCNL group compared to the fURL group (RR: 1.26; 95% CI: 0.82, 1.95; *P* = 0.29; Fig. 5a).

#### Perforation

Meta-analysis of 6 studies [6, 11, 14, 21–23] by a fixed effects model showed the occurrence rate of pelvis or ureter perforation was not significantly different in the PCNL group compared to the fURL group (RR: 1.11; 95% CI: 0.38, 3.25; *P* = 0.84; Fig. 5b).

#### Requiring drug analgesia

Meta-analysis of 5 studies [6, 9, 14, 15, 18] by a random effects model showed the rate of requiring drug analgesia after surgery was not significantly different in the PCNL group compared to the fURL group (RR: 1.23; 95% CI: 0.59, 2.55; *P* = 0.58; Fig. 5c).

#### Publication Bias

Begg’s test and Egger’s test were used to assess the publication bias of those studies. Funnel plot showed that there were no significant publication bias observed among those studies. As showed in Table 3, all of Begg’s *p* value and Egger’s p value were not significant.

**Table 3:** Results of publication bias testing

Study project	Included study	Begg testing		Egger testing		95% CI
		***z***	***P***	***t***	***P***	
Stone free rate	19	1.19	0.234	1.21	0.244	−0.750, 2.750
Operative time	14	0.11	0.913	1.51	0.156	−2.887, 16.001
Decline of Hb	7	1.5	0.133	−2.13	0.101	−5.691, 0.756
Blood transfusion	6	0.24	0.806	0.84	0.463	−1.288, 2.208
Bleeding/hematuria	9	0.73	0.466	1.36	0.216	−0.934, 2.204
Fever/infection	12	0.89	0.373	−1.22	0.251	−2.639, 0.774
Perforation	6	0.38	0.707	−0.6	0.578	−8.271, 5.314
Requiring drug analgesia	5	0.24	0.806	0.56	0.615	−5.318, 7.599

#### Sensitivity analysis

Sensitivity analysis was conducted to determine the influence of each eligible study on the overall outcome and test the robustness of all results above. When any individual study was excluded, the pooled result was not significantly changed, showing the robustness of the results.

## Discussion

Both PCNL and fURL are important methods for the treatment of upper urinary calculi. Our paper uses the meta-analysis method to comprehensively summarize the results of PCNL and fURL clinical studies to increase the sample size and improve the statistical power. According to the inclusion criteria and exclusion criteria, 19 studies were included, a total of 1822 cases of upper urinary calculi. In our paper, eight representative outcome indicators were selected to evaluate the efficacy and safety of the two surgical procedures.

Calculus clearance rate is the most important outcome measure for evaluating the effectiveness of PCNL and fURL in treating upper urinary calculi. Stone clearance is defined as follow-up 1 ~ 3 months after the operation, the last imaging examination (X-ray, ultrasonography, or CT), no residual stone or residual stone less than 4 mm without clinical symptoms was successful. In our paper, the combined analysis of the stone clearance rates of 19 studies showed that PCNL stone clearance rate is higher than fURL (RR:1.07, 95% CI: 1.03, 1.12). Davis [27] et al. reported in their meta-analysis the stone clearance rate of mPCNL was higher than that of fURL, and the results were consistent with us. The efficacy of partial fURL in the treatment of large areas of kidney stones [28, 29] reported that stone size is the main parameter affecting the success rate of fURL. The success rate of fURL may be affected as the size of the stone changes. However, in the study by Davis [27] et al., the stone size was subgrouped separately, and the stone clearance rate of mPCNL was higher in the kidney stone group > 2 cm or in the kidney stone group < 2 cm fURL, therefore, whether the two surgical stone removal rate is different due to the size of the stone is still controversial, we hope there is further evidence-based medical evidence.

The operation time is an indirect indicator of the patient undergoing surgery and anesthesia stress injury, and is related to the experience of surgeons and hospital equipment [15]. Some studies have reported that fURL surgery is longer than PCNL [10, 16, 20, 22, 26], but in the comparison of the two surgical procedures, the time of PCNL operation was similar to fURL, and the difference was not statistically significant (WMD: − 2.84 min; 95% CI: − 12.91, 7.23; *P* = 0.58).

Complications of upper urinary calculi surgery include renal collecting system or ureteral perforation and laceration, near organ injury, bacteremia, toxemia, infection, fever, intraoperative or postoperative bleeding, ureteral stricture, urine leaks, etc. The incidence of PCNL complications increases with the diameter of the working channel [10, 16, 20, 22, 26, 30]. It has been reported that mPCNL (according to the working channel ≤20) has a similar stone clearance rate and fewer complications than the standard channel PCNL (working channel = 30) [22, 31]. Based on the complication data provided by the 19 articles included in this article, the amount of Hb decreased before and after surgery, the proportion of patients requiring blood transfusion, postoperative hemorrhage or hematuria, infection or fever, postoperative analgesic drug use rate, treatment of upper urinary calculi complications of pelvic or ureteral perforation were analyzed separately to understand the complications of PCNL and fURL in the treatment of upper urinary calculi.

The amount of Hb decreased, blood transfusion, bleeding or hematuria are important for evaluating the safety of surgery. In terms of the amount of Hb decreased before and after surgery, it can be seen that the amount of Hb decreased before and after PCNL surgery was more than fURL, suggesting that the amount of bleeding in PCNL was more (WMD: 1.07; 95% CI: 0.54, 1.61; *P* < 0.0001). In terms of blood transfusion, it can be seen that PCNL requires more blood transfusion than fURL, suggesting that PCNL has more intraoperative and postoperative bleeding (RR: 5.04; 95% CI: 1.78, 14.24; *P* = 0.002). Postoperative bleeding or hematuria, suggesting that the incidence of postoperative bleeding after PCNL is higher than fURL (RR: 2.72; 95% CI: 1.55, 4.75; *P* = 0.0005). The decrease of Hb before and after surgery, the need for blood transfusion and postoperative hemorrhage or hematuria showed that PCNL had greater damage than fURL and more bleeding. The reason for the analysis may be that the kidney is rich in blood supply, and PCNL needs to puncture the kidney to establish a working channel. During the puncture and operation, it is easy to damage the interstitial blood vessels of the renal parenchyma, resulting in more intraoperative blood loss, and the injured renal blood vessels develop arteriovenous veins after operation. A arteriovenous fistula or pseudoaneurysm is a well known source of postoperative bleeding from PCNL [32]. After the operation, the renal fistula is retained, and the stimulation of the fistula can also cause renal vascular rupture. And fURL does not need to be established because it goes retrograde along the physiological channel into the lesion. As a channel, the damage to the body is small, the amount of bleeding is small, there is no need to place the fistula after operation, there is no continuous stimulation, and compared with PCNL, the complications of bleeding during fURL are strictly performed in the normalized operation, can be prevented [5].

Our performed a meta-analysis for the number of painful cases requiring painkillers after surgery, postoperative infection or fever, and the incidence of intraoperative pelvic or ureteral perforation. For the classification of pain, most studies used VAS visual scores [6, 22, 23], but this method is subjective. The use of analgesic drugs is associated with hospital policies, doctors’ experience, patient’s appeals, so there is heterogeneity. It can be seen that there is no statistical difference in the number of cases requiring painkillers and analgesia after PCNL and fURL (RR: 1.23; 95% CI: 0.59, 2.55; *P* = 0.58). There was no significant difference in the incidence of postoperative infection or fever between PCNL and fURL (RR: 1.26; 95% CI: 0.82, 1.95; *P* = 0.29). There was no significant difference in the incidence of pelvic or ureteral perforation between the two surgical procedures (RR: 1.11; 95% CI: 0.38, 3.25; *P* = 0.84).

In summary, PCNL and fURL have advantages and disadvantages in the treatment of upper urinary calculi. PCNL has higher stone clearance rate than fURL, and fURL has the advantage of less intraoperative/postoperative bleeding. Therefore, the appropriate surgical method should be selected according to the specific conditions of the patient, the experience of the doctor, and the conditions of the hospital.

## Conclusion

In the treatment of upper urinary tract stones, the stones clearance rate of PCNL is higher than fURL. The Decline of HB, the number of blood transfusion, the incidence of postoperative bleeding or hematuria in PCNL group was significantly high than that of fURL group, so the safety of fURL is higher than PCNL. Therefore, appropriate surgical methods should be selected according to different situations.

## Data Availability

The datasets used and/or analysed during the current study are available from the corresponding author on reasonable request.

## Abbreviations

ESWL: Extracorporeal shock wave lithotripsy
RIRS: Retrograde ureteroscopic lithotripsy
PCNL: Percutaneous nephrolithotomy
mPCNL: Microchannel PCNL
UMP: Ultra-fine passage percutaneous kidney mirror lithotripsy
SMP: Ultra-microchannel percutaneous nephrolithotomy
fURL: Ureteroscopy
AUA: The American Urological Association
PRISMA: The Preferred Reporting Items for Systematic Reviews and Meta-analysis
CNKI: China National Knowledge Infrastructure databases
Hb: hemoglobin
NOS: The Newcastle-Ottawa Scale
RCTs: Randomized controlled trials
95% CI: 95% confidence intervals
